# Five-Year Progression of Refractive Errors and Incidence of Myopia in School-Aged Children in Western China

**DOI:** 10.2188/jea.JE20140258

**Published:** 2016-07-05

**Authors:** Wen-Jun Zhou, Yong-Ye Zhang, Hua Li, Yu-Fei Wu, Ji Xu, Sha Lv, Ge Li, Shi-Chun Liu, Sheng-Fang Song

**Affiliations:** 1 Department of Ophthalmology, Yongchuan Hospital, Chongqing Medical University, Chongqing, People’s Republic of China; 2 Statistics Department, Chongqing Medical University, Chongqing, People’s Republic of China

**Keywords:** refractive errors, myopia, school-aged child, longitudinal cohort study

## Abstract

**Background:**

To determine the change in refractive error and the incidence of myopia among school-aged children in the Yongchuan District of Chongqing City, Western China.

**Methods:**

A population-based cross-sectional survey was initially conducted in 2006 among 3070 children aged 6 to 15 years. A longitudinal follow-up study was then conducted 5 years later between November 2011 and March 2012. Refractive error was measured under cycloplegia with autorefraction. Age, sex, and baseline refractive error were evaluated as risk factors for progression of refractive error and incidence of myopia.

**Results:**

Longitudinal data were available for 1858 children (60.5%). The cumulative mean change in refractive error was −2.21 (standard deviation [SD], 1.87) diopters (D) for the entire study population, with an annual progression of refraction in a myopic direction of −0.43 D. Myopic progression of refractive error was associated with younger age, female sex, and higher myopic or hyperopic refractive error at baseline. The cumulative incidence of myopia, defined as a spherical equivalent refractive error of −0.50 D or more, among initial emmetropes and hyperopes was 54.9% (95% confidence interval [CI], 45.2%–63.5%), with an annual incidence of 10.6% (95% CI, 8.7%–13.1%). Myopia was found more likely to happen in female and older children.

**Conclusions:**

In Western China, both myopic progression and incidence of myopia were higher than those of children from most other locations in China and from the European Caucasian population. Compared with a previous study in China, there was a relative increase in annual myopia progression and annual myopia incidence, a finding which is consistent with the increasing trend on prevalence of myopia in China.

## INTRODUCTION

It is well known that refractive error, especially myopia, is a common cause of visual impairment in children.^1^^,^^2^ In the past few decades, numerous cross-sectional studies have provided information on the pattern of prevalence and risk factors for myopia in children.^3^^–^^21^ These studies have shown that the prevalence of refractive error varies widely, depending on geography, ethnicity, sex, and age. Generally, the prevalence of myopia has been reported to be higher among East Asians and those living in urban locations, such as Singapore, Hong Kong, Taiwan, Japan, and Korea, than among European Caucasians and those living in rural areas.^16^^,^^17^^,^^22^^–^^25^ In China, previous population-based surveys have also shown that the prevalence of myopia was higher than in other countries, such as Nepal, Chile, and India.^3^^,^^4^^,^^7^^,^^26^^–^^28^ Several recent studies carried out in northern China and eastern China have shown that the prevalence of myopia still appears to be increasing, and in particular, that the prevalence of high myopia is increasing even more markedly.^29^^–^^32^ A study of university students in Shanghai, China, revealed that more than 95% of the study populations were myopic, and that about 10–20% were highly myopic (myopia refractive error >−6 D).^33^

Although there is an abundance of cross-sectional refractive data for school-aged children, there have been relatively few longitudinal studies in this age group, even though measuring the incidence of myopia is essential to determine differences in risk between populations. In mainland China, there has been only one longitudinal study of refractive error, which was carried out in Shunyi District in 2000 and reported an annualized incidence of myopia of 7.8% and a rate of myopic progression of −0.17 D per year.^34^

In 2006, we carried out a population-based cross-sectional survey of refractive error in children aged 6 to 15 years in Yongchuan District of Chongqing City, Western China.^35^ Here, we conducted a 5-year longitudinal follow-up study and reported the change in refractive error and the incidence of myopia with age in those children.

## METHODS

### Population

From October 2006 to January 2007, 3070 children were examined in a population-based cross-sectional survey of refractive error in Yongchuan District, one of 40 administrative districts in Chongqing City. According to the China Sixth National Population Census, which was conducted in 2010, the municipality of Chongqing has a population of 28.85 million and is considered an economic and cultural center of Western China. Yongchuan District was chosen for the study because it had a relatively stable population (1.02 million, +0.90% annual average growth rate from the 2010 Census), with its socioeconomic status being ranked in the middle of cities in Western China and most residents in this district being Han Chinese.

The original study sample of children aged 6 to 15 years was selected randomly using cluster sampling of geographical residential areas, namely residence administrative communities (RACs) and villages, throughout Yongchuan District. There were 93 RACs and 631 villages in Yongchuan District. For cluster sampling, RACs and villages with large populations were further divided, and those with small populations were combined to create clusters with an estimated 100 to 150 eligible children each. There were 78 clusters that met the study criteria, and 28 were randomly selected for the study, including 6 from urban areas, 13 from rural areas, and 9 from suburban areas. In the 28 selected clusters, children meeting the following criteria were recruited: age 6 to 15 years at examination; informed consent form signed by parents or legal guardians; with cycloplegic dilation in both eyes; and no history of cardiovascular or nervous system diseases, including congenital heart diseases, hypoxic ischemic encephalopathy, and cognitive impairment. Children were excluded if they had eye injuries or eye diseases; if they had a history of untreated closed-angle glaucoma or untreated anatomically narrow angles; if they were allergic to any ingredient in 1% cyclopentolate solution; if they could not fix their gaze during testing; or if they refused to continue the examinations due to eye discomfort during cyclopentolate administration. As previously reported, distribution pattern of refractive status, prevalence of refractive error, and possible environmental factors were determined.^35^

The longitudinal follow-up of children commenced in November 2011 and continued through March 2012. Before the initiation of fieldwork in the longitudinal follow-up survey, we visited each of the 28 original sample RACs and villages to obtain updated information on all children originally examined. Using official RACs and village registers, demographic information was gathered, including changes of address for children who had relocated outside of the district and names of the current school for children still attending district schools.

**Figure 1.**
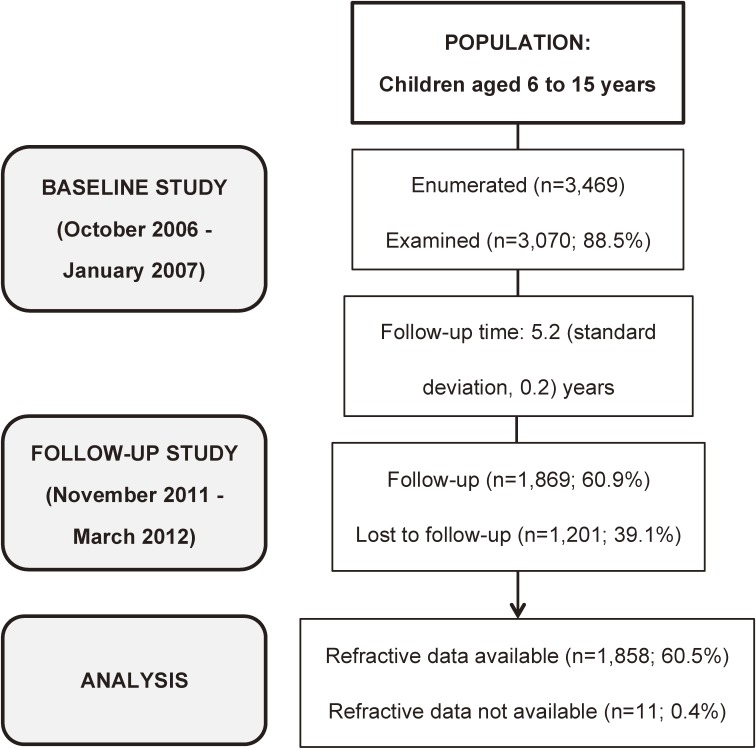
Flowchart of the study examination procedures, showing the subjects available for analysis in the baseline and follow-up study.

After gathering demographic information, the follow-up examination was carried out at the child’s school for most children. For children who had moved out of the district schools or already finished their studies at the time of survey, the follow-up examination was also performed in a door-to-door way if contact was possible. The inclusion and exclusion criteria of the baseline survey were also used in the follow-up survey. Details of the subjects examined in the baseline and follow-up study are shown in Figure 1.

### Procedures

Human subject research approval for the study protocol was obtained from the World Health Organization (WHO)’s Secretariat Committee on Research Involving Human Subjects. The study protocol was also approved by the Human Research Ethics Committee of Chongqing Medical University. The protocol adhered to the provisions of the Declaration of Helsinki for research. Informed written consent was obtained from parents or from participants who were over the legal age of consent (18 years) before participation. The Bureau of Education and Bureau of Health in Yongchuan District approved the implementation of this study.

Eye examinations were performed by a medical team consisting of three ophthalmic nurses, two ophthalmologists, and two optometrists. The examination process began with testing visual acuity at 4 m using the “Early Treatment of Diabetic Retinopathy study” (ETDRS) LogMAR visual acuity chart (Precision Vision, La Salle, IL, USA). This was followed by ocular motility evaluation, anterior segment examination, and autorefraction with a hand-held Nikon Retinomax K-Plus (Nikon, Tokyo, Japan). After ensuring that there was no risk for a medical mydriasis, both pupils were dilated with two drops of cyclopentolate 1% administered 5 minutes apart, and the pupillary light reflex was checked 20 min later. If the pupillary light reflex was still present, a third drop was administered. Cycloplegia was considered complete if the pupil dilated to 6 mm or greater and light reflex was absent. After cycloplegic dilation, the team ophthalmologist took a second autorefraction measurement. Each eye was measured at least 3 times. The difference between the maximum and minimum value of the measurements of spherical refractive error and cylindrical refractive error had to be less than 0.5 D; otherwise the measurements had to be repeated. The examination process was finalized with the fundus examination using a direct ophthalmoscope (YZ6E; Six Six Vision Corp., Suzhou, China).

### Definitions

As previous studies have described,^36^^,^^37^ refractive status was determined by the spherical equivalent refraction (SER) of both eyes (calculated as sphere + 1/2 cylinder). Myopia was defined as a SER of ≤−0.50 D in one or both eyes and hyperopia as a SER of ≥+0.50 D in one or both eyes. Astigmatism was defined as ≥1.00 D cylinder refraction in one or both eyes. Emmetropia was defined as a SER of >−0.50 to <+0.50 D in both eyes. Refractive error was further subdivided for analysis into moderate myopia (≤−3.00 D), mild myopia (≤−0.50 to >−3.00 D), emmetropia (>−0.50 to <+0.50 D), mild hyperopia (≥+0.50 to <+2.00 D), and significant hyperopia (≥+2.00 D).

Cumulative shift in refractive error in each eye was determined by the difference in mean SER between baseline and follow-up measures (the follow-up measurement minus the original baseline measurement). Annual shift in refraction was the difference in mean SER divided by the mean follow-up time in years. Cumulative incidence of myopia was defined as the proportion of children who were not myopic (initial emmetropes and hyperopes) at baseline but who subsequently developed myopia during the follow-up period. The annual incidence rates were calculated by dividing the cumulative percentage by the mean follow-up time in years.

### Statistical analyses

Data were analyzed using an SPSS software program (SPSS for Windows, Rel.13.0.0.2004; SPSS, Chicago, IL, USA). Since the refraction distributions of left eyes and right eyes were similar (Pearson coefficient = 0.90) and the data from left eyes had fewer outliers, only the data from left eyes are presented in this report.

Multiple logistic regression was used to investigate the association of sex, age, and the amount of refractive error at baseline with myopic progression of refractive error and myopia incidence. In multiple logistic regression modeling, sex, age, and the amount of refractive error at baseline were considered as covariates, while myopic progression and myopia incidence were considered as binary outcome variables. Myopic progression means a progression of refractive error in a myopic direction. As autorefractors round up refractive measures to the nearest 0.12 D in either direction, there is a possibility of measurement error of 0.25 D at each point of examination. Only shifts in refraction of ≥0.50 D during the follow-up period were considered clinically significant. Thus, myopic progression was defined using progression thresholds of −0.50 D or −1.00 D in this study. For the other outcome variable, myopia incidence refers to the event in which a participant who was not myopic (SER >−0.50 D in one or both eyes) subsequently developed myopia (SER ≤−0.50 D in one or both eyes) during the follow-up period.

Odds ratios (ORs) and 95% confidence intervals (CIs) were calculated. All *P*-values were two-sided and were considered statistically significant when the values were less than 0.05.

## RESULTS

### Characteristics of the study population

Table 1 shows the demographic makeup of the study population in the baseline and follow-up surveys. In the original 2006 survey, a total of 3469 children between 6 and 15 years of age were enrolled, and 3070 were examined. At the follow-up study in 2011, 1869 children from the original sample were reexamined, and 1201 children were lost to follow-up. Of those who were reexamined at the follow-up, 896 (47.9%) were male and 973 (52.1%) were female. The mean (standard deviation [SD]) time between baseline and follow-up examinations was 5.2 (0.2) years. The percentage of baseline examinees with follow-up examinations was 60.9% overall, with age-specific rates ranging from 74.5% to 40.9%. Of the children who were reexamined, 1858 had complete refraction data at follow-up available for longitudinal analysis. Eleven of the 1869 children with follow-up examinations were excluded from refractive error analyses because of ocular pathology or inadequate cycloplegic dilation.

**Table 1.** Subjects enrolled, examined, and lost to follow-up by baseline age

Baseline age (years)	Number (%) enrolled at baseline	Number (%) examined		Number (%) lost to follow-up	Examination follow-up percentage

		Baseline	Follow-up		
6	300 (8.65)	239 (7.79)	178 (9.52)	61 (5.08)	74.48
7	362 (10.44)	313 (10.20)	221 (11.82)	92 (7.66)	70.61
8	369 (10.64)	339 (11.04)	240 (12.84)	99 (8.24)	70.80
9	378 (10.90)	350 (11.40)	239 (12.79)	111 (9.24)	68.29
10	373 (10.75)	341 (11.12)	227 (12.15)	114 (9.49)	66.57
11	349 (10.06)	319 (10.39)	216 (11.56)	103 (8.58)	67.71
12	358 (10.32)	305 (9.93)	168 (8.99)	137 (11.41)	55.08
13	325 (9.37)	285 (9.28)	136 (7.28)	149 (12.41)	47.72
14	379 (10.93)	354 (11.53)	152 (8.13)	202 (16.82)	42.94
15	276 (7.96)	225 (7.33)	92 (4.92)	133 (11.07)	40.89
All	3469 (100)	3070 (100)	1869 (100)	1201 (100)	60.88

In logistic regression modeling to investigate the influence of baseline refractive error, age, and sex on follow-up success, we found that those with younger age, female sex, and emmetropia at baseline were more likely to have a follow-up examination.

### Change in refraction and progression of myopia

Change in spherical equivalent refraction during the follow-up interval ranged from −10.00 D to +4.50 D, with a mean (SD) of −2.21 (1.87) D for all age cohorts. The 95% CI around this estimate of the mean was −2.13 D to −2.29 D. This represented an annual shift of refraction in a myopic direction of −0.43 D. There were 84 children with progression to significant myopia of −6.00 D or more over the follow-up interval. Among cases with hyperopic shift (those becoming more positive), 10 children had change of more than +1.00 D.

**Figure 2.**
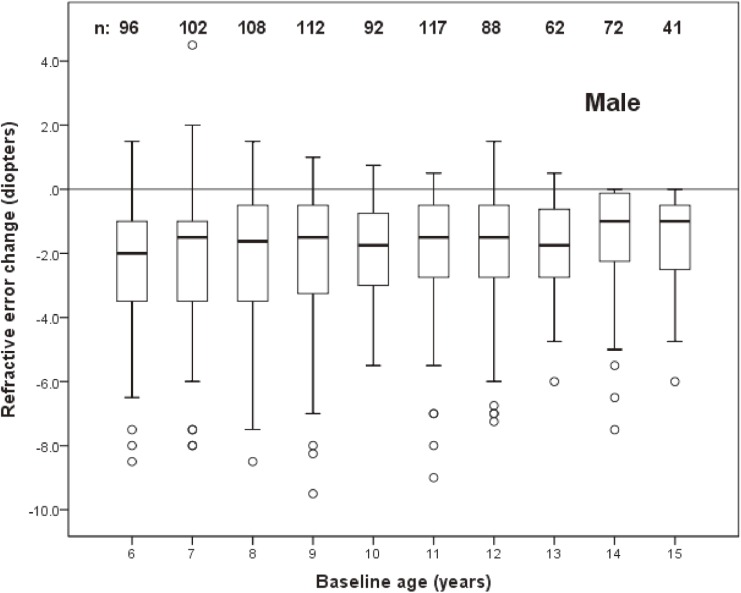
Box plot representations of the distribution of change in spherical equivalent refractive error of male subjects as a function of age at baseline. The box extends from the 25th to the 75th percentile, the interquartile range, with the bar inside each box representing the median. The whiskers extend to the lower and upper extremes, defined as the 25th percentile minus 1.5 times the interquartile range and the 75th percentile plus 1.5 times the interquartile range. Sample sizes for each age group are shown.

**Figure 3.**
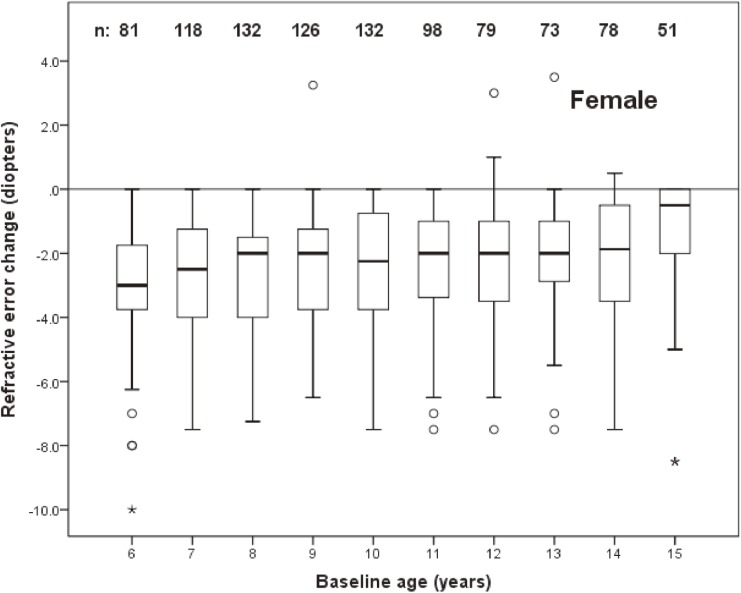
Box plot representations of the distribution of change in spherical equivalent refractive error of female subjects as a function of age at baseline. The box extends from the 25th to the 75th percentile, the interquartile range, with the bar inside each box representing the median. The whiskers extend to the lower and upper extremes, defined as the 25th percentile minus 1.5 times the interquartile range and the 75th percentile plus 1.5 times the interquartile range. Sample sizes for each age group are shown.

The distribution of change in refractive error by age at baseline in left eyes of males and females are shown in Figure 2 and Figure 3, respectively. The mean (SD) change was −1.99 (1.88) D in males (95% CI, −1.87 D to −2.11 D) and −2.41 (1.83) D in females (95% CI, −2.29 D to −2.53 D).

**Figure 4.**
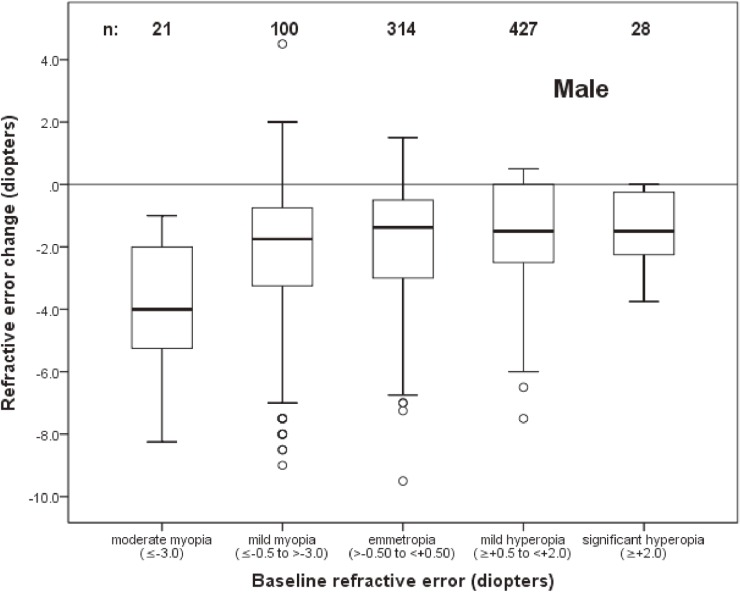
Box plot representations of the distribution of change in spherical equivalent refractive error of male subjects as a function of refractive error at baseline. Sample sizes for each baseline refractive error group are shown. (See Figure 2 caption for an explanation of box plots.)

**Figure 5.**
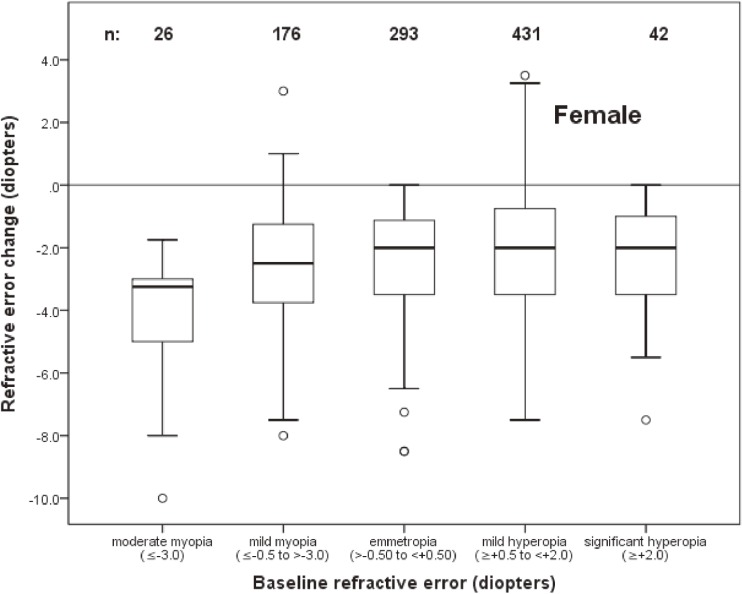
Box plot representations of the distribution of change in spherical equivalent refractive error of female subjects as a function of refractive error at baseline. Sample sizes for each baseline refractive error group are shown. (See Figure 2 caption for an explanation of box plots.)

The distribution of change as a function of refractive status at baseline among males and females is shown in Figure 4 and Figure 5, respectively. The mean change in baseline myopic eyes (refractive error of at least −0.50 D) was −3.56 D versus −1.32 D in all other eyes.

The association of change in refractive error with age, sex, and refractive error at baseline was investigated using logistic regression. Because of the nonlinear relationship between baseline refractive error and refractive error change (Figure 4 and Figure 5), eyes with a baseline spherical equivalent refractive error of ≤0.0 D were modeled separately from those with refractive error of >0.0 D. Refractive error status at baseline was modeled as a continuous variable.

For eyes with baseline refractive error of ≤0.0 D, myopic progression of at least −0.50 D was associated with younger age (odds ratio [OR] 1.27; 95% CI, 1.12–1.34), female sex (OR 1.68; 95% CI, 1.32–2.38), and higher myopic refractive error at baseline (OR 1.73; 95% CI, 1.35–2.21). When only eyes that were myopic (≤−0.50 D) at baseline were modeled, myopic progression of at least −0.50 D was associated with higher myopic refractive error at baseline (OR 1.45; 95% CI, 1.12–1.84) and female sex (OR 1.43; 95% CI, 1.16–1.71), but the association with age (OR 1.01; 95% CI, 0.93–1.12) was no longer statistically significant.

Upon increasing the myopic progression threshold to −1.00 D, younger age (OR 1.21; 95% CI, 1.10–1.32), female sex (OR 1.43; 95% CI, 1.24–1.83), and higher myopic refractive error at baseline (OR 1.56; 95% CI, 1.34–2.07) remained statistically significant for eyes with baseline refractive error ≤0.0 D.

In modeling eyes with refractive error of >0.0 D at baseline, myopic progression of at least −0.50 D was associated with younger age (OR 1.15; 95% CI, 1.04–1.23), female sex (OR 1.72; 95% CI, 1.49–2.16), and higher hyperopic refractive error at baseline (OR 1.34; 95% CI, 1.16–1.73).

With a change of at least −1.00 D, the associations with younger age (OR 1.13; 95% CI, 1.07–1.25) and female sex (OR 1.64; 95% CI, 1.43–2.21) remained significant, but higher hyperopic refractive error at baseline (OR 0.97; 95% CI, 0.89–1.16) was no longer statistically significant.

Table 2 shows the number of children with astigmatic error and astigmatism change between baseline and follow-up. Almost 98% of children had cylindrical measurements of less than 1.00 D at both baseline and follow-up. Within the entire study population, the magnitude of astigmatic error showed little change over the 5.2-year period: an estimated mean (SD) decrease of 0.03 (0.23) cylindrical D. In logistic regression modeling, we found that astigmatism change (an increase of ≥0.50 D) was not associated with age, sex, or refractive error at baseline.

**Table 2.** Number (%) of children with astigmatic error and astigmatism change between baseline and follow-up

	Astigmatic error				

	0.0–0.50	0.75–1.0	1.25–1.75	≥2.0	All
Baseline	1617 (87.0)	184 (9.9)	45 (2.4)	12 (0.6)	1858 (100)
Follow-up	1744 (93.9)	86 (4.6)	21 (1.1)	7 (0.4)	
Astigmatism change	0.03 cylindrical diopter (±0.23)				

### Incidence of myopia

Table 3 compares the ametropic status of children at baseline with that at follow-up. Of the 1591 children who were not myopic at baseline (emmetropes and hyperopes), 874 had become myopic by follow-up, resulting in a cumulative incidence of 54.9% (95% CI, 45.2%–63.5%) and an annual incidence of 10.6% (95% CI, 8.7%–13.1%). Of the children who were significantly hyperopic at baseline, 66.1% were no longer hyperopic at follow-up, with an annual decline of 12.7%.

**Table 3.** Number (%) of children with ametropia at baseline and follow-up

	Follow-up			

	Hyperopes	Emmetropes	Myopes	All
Baseline				
Hyperopes	19 (33.9)	17 (30.4)	20 (35.7)	56 (3.0)
Emmetropes	2 (0.13)	679 (44.2)	854 (55.6)	1535 (82.6)
Myopes	0 (0.0)	53 (19.9)	214 (80.1)	267 (14.4)
All	21 (1.1)	749 (40.3)	1088 (58.6)	1858 (100)

**Figure 6.**
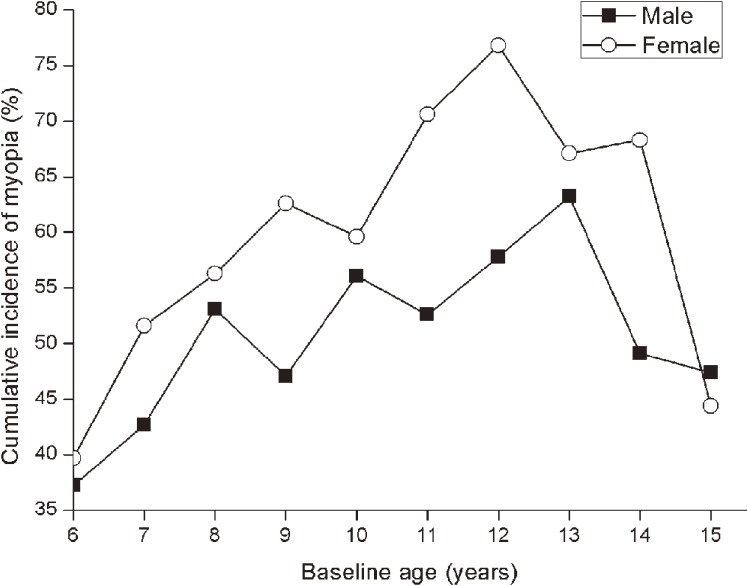
Age-specific cumulative 5.2-year incidence of myopia among male and female emmetropes/hyperopes as a function of age at baseline.

The cumulative incidences of myopia by age and gender are shown in Figure 6. Highest incidences were found in 13-year-old boys (63.2%) and 12-year-old girls (76.8%), with annual incidences of 12.2% and 14.8%, respectively. Incidence was lowest among children who were 6-year-olds at baseline: 37.2% (95% CI, 23.2%–53.2%) for males and 39.7% (95% CI, 21.6%–57.7%) for females, with annual incidences of 7.2% and 7.6%, respectively. The cumulative incidence for children of all ages was 50.2% for males and 59.5% for females. The cumulative incidence of myopia for the study population as a whole was 54.9%, with annual incidence of 10.6% (as noted above). In logistic regression modeling of myopia incidence with age and sex as covariates, we found that females had significantly greater odds of incident myopia (OR 1.55; 95% CI, 1.13–1.84) and that increasing age was positively correlated with increased incidence of myopia (OR 1.21; 95% CI, 1.04–1.42).

## DISCUSSION

To our knowledge, this is the first longitudinal study examining the incidence and progression of refractive error in school-aged children in Western China. The children in our sample were selected randomly from all over the Yongchuan District of Chongqing City and were all Chinese Han ethnicity. As anticipated, there was an overall myopic progression in refractive error as measured by cycloplegic autorefraction between the baseline and follow-up examinations. The 5-year cumulative mean rate of progression of myopia in our study was −2.21 D, with an annual progression of refraction in a myopic direction of −0.43 D. This myopic progression in refractive error was associated with younger age, female sex, and higher refractive error at baseline (either myopic or hyperopic error).

There have been several population-based longitudinal studies in different geographical and ethnic contexts that have reported the incidence and progression of refractive error in school-aged children. In Hong Kong, the average rate of myopic progression has been reported to be −0.40 D per year in Chinese children aged 5 to 16 years.^22^ This annual progression rate is very close to that of our population. The Singapore Cohort study Of the Risk factors for Myopia (SCORM) reported 3-year cumulative mean myopia progression rates of −2.40 D in 7-year-olds, −1.97 D in 8-year-olds, and −1.71 D in 9-year-olds, with respective annual progression rates of −0.80 D, −0.66 D, and −0.57 D.^38^ The age-matched annual progression rates in our study were −0.47 D in 7-year-olds, −0.44 D in 8-year-olds, and −0.41 D in 9-year-olds, which are lower than those in Singapore. In mainland China, a lower rate of myopic progression of −0.17 D per year was reported in children aged 5 to 13 years in Shunyi.^34^ In the United States, the Correction of Myopia Evaluation Trial report an annual progression of myopia of −0.50 D in the control group of children aged 6 to 11 years at baseline.^39^ An annual progression rate of −0.38 D was reported from the ethnically diverse Collaborative Longitudinal Evaluation of Ethnicity in Refractive Error population of children with a mean age of 9 years at baseline.^40^ In Australian schoolchildren, a recent study reported annual progression rates of −0.16 D in 7-year-olds and −0.15 D in 12-year-olds.^36^ The annual rate reported in these studies has varied a lot, possibly because of variations in geography, ethnicity, and environment. The annual progression rate of myopia tends to be higher in East Asians than that in European Caucasians, which is consistent with the higher prevalence of myopia reported in East Asians. The study in Australian children showed that children of East Asian ethnicity had almost 3 times the odds of a significant shift in refraction as European Caucasian children.^36^ Children in urban areas of East Asia, such as Hong Kong and Singapore, tend to be more likely to have a myopic shift in refraction than children in rural areas of China, such as Shunyi. The Shunyi study, carried out 12 years before, evaluated children of similar age and ethnicity as those in our study. However, to some extent, there exists some environmental and geographical differences between Shunyi and the Yongchuan district. Shunyi County is located 30 kilometers northeast of Beijing City, away from the urban area and mainly on a plain. In Shunyi, 97% of the population lived in rural areas at the time of survey. Children recruited in the Shunyi study were all from rural areas. The Yongchuan district, which lies mainly on hilly land, is located west of Chongqing City. One-third of its populations live in urban areas, and the socioeconomic status and living standards are higher than those in Shunyi 12 years ago. The children in our study included those from urban, rural, and suburban areas.

In our study, the annual progression rate of myopia was much higher than that in Shunyi. Although the environmental and geographical factors may have an influence on the progression of myopia, our results suggest that the progression of myopia in China may be increasing. This is consistent with the increasing prevalence of myopia reported in China, which may be due to the rapid socioeconomic development of China. We found myopic progression to be associated with younger age, which is counter to the finding of higher myopic progression among older children in the Shunyi study but consistent with the findings in Hong Kong and Singapore. Our finding that myopia progression was also associated with female sex and higher levels of baseline myopia replicated findings from the previous reports.^22^^,^^38^ The studies in Shunyi and Australia have also reported an increased myopic progression in refraction for children with baseline hyperopia.^34^^,^^36^

We found that the 5-year cumulative incidence of myopia across all ages and both sexes was 54.9%, with an annual incidence of myopia of 10.6%. Our annual incidence of myopia was lower than that in Hong Kong and Singapore^22^^,^^38^ but higher than that in Shunyi.^34^ The Hong Kong study reported an annual incidence of 14.4% in children aged 5 to 16 years at baseline. In Singapore, a similar incidence was reported (14.2%); however, this was in children whose baseline age ranged from 7 to 9 years. In a similarly aged group, Zhao et al reported a lower annual incidence of 7.8% in Shunyi. In comparison with studies of other predominantly European Caucasian populations, children of East Asian ethnicity had a greater incidence of myopia. The Orinda Longitudinal Study of Myopia in the United States reported an annual incidence of myopia of 4.3% in children aged approximately 8 years at baseline, which is much lower than the incidence among our children aged 8 years at baseline (10.5%).^41^ In Australia, the annual incidence of myopia was 2.2% in 7-year-olds and 4.1% in 12-year-olds at baseline.^36^ This study also found children of East Asian ethnicity had a higher annual incidence of myopia than European Caucasian children. We found a higher incidence of myopia among girls, with an OR of 1.55 (95% CI, 1.13–1.84), which was consistent with previous studies.^22^^,^^34^^,^^38^ A Finnish study also reported greater myopic progression in girls.^42^ One possible explanation is that girls tend to read and write more, at least at the primary-school level. The subsequent increase in near-work predisposes them to myopia development.

It should be noted that not all children in the original study were included in the follow-up study. These were primarily older children who had completed schooling and had left the Yongchuan area for work in nearby Chongqing or other cities in China. Although we tried to improve the participation rate by conducting a door-to-door examination for children who migrated out of the district for work or education if contact was possible, there were many older children still lost. This resulted in relatively low participation rates of 47.7%, 42.9%, and 40.9% in 13-, 14-, and 15-year-olds at baseline, respectively. Findings pertaining to these age groups are therefore subject to potentially significant bias. In these three age groups, it was found that children from urban areas and children still attending school were more likely to have a follow-up examination. Because those children would experience more near-work activities and intense schooling, which would result in increasing myopic progression, refractive error change for these age groups might have been overestimated. However, the incidence of myopia in these age groups might be underestimated, since those children vulnerable to myopic development are likely to have been myopic at baseline.

For all age groups, we found that those with younger age, female sex, and emmetropia at baseline were more likely to have a follow-up examination in logistic regression modeling. Because those with younger age and female sex were found to have comparatively large changes in refractive error, it is possible that refractive error change for the study population as a whole was slightly overestimated. In addition, to investigate the effect of environmental factors on the prevalence of refractive errors, our original study showed a significant relationship between school type and the prevalence of myopia. Academically challenging schools had more myopic children than the regular schools. To explain this finding, we added up school students’ average reading and writing times based on course schedule, counseling after class, and homework time. Our investigation showed that children in academically challenging schools spent more time reading and writing than those in regular schools. Our findings indicated that near-work activity may contribute to the development of myopia. However, we did not include environmental factors as risk factors for myopic progression in the follow-up study, since the time each child spent in reading and writing varied over the long follow-up period. Some children were going to enter academically challenging schools after regular school, while others had already graduated at the time of survey.

In conclusion, the progression and incidence of myopia in school-aged children in the world vary widely, depending on geography, ethnicity, sex, and age. We reported an annual myopia progression of −0.43 D and an annual myopia incidence of 10.6% in the Yongchuan District of Chongqing City, Western China. The annual myopia progression and annual myopia incidence were higher than in European Caucasian children. Compared with the Shunyi study, which was carried out in another part of China 12 years before our study, there was a relative increase in annual myopia progression and annual myopia incidence. This may be the result of the highly competitive education system accompanying the rapid socioeconomic development of China. To revitalize the country through science and education, the Chinese government implemented 9-year compulsory education in 1986. Subsequently, the government made constant efforts to deepen the reform of the educational system. Now, China’s school education system includes pre-school, primary school, secondary school, high school, and university, as well as graduate school.^43^ Getting into university is highly competitive, which means children pursuing university study undertake more near-work activities and intense schooling. It is likely that both the rate and severity of myopia will increase over time in China.
